# X-ray magnetic diffraction under high pressure

**DOI:** 10.1107/S2052252519007061

**Published:** 2019-06-21

**Authors:** Yishu Wang, T. F. Rosenbaum, Yejun Feng

**Affiliations:** a The Institute for Quantum Matter and Department of Physics and Astronomy, The Johns Hopkins University, Baltimore, Maryland 21218, USA; b Division of Physics, Mathematics, and Astronomy, California Institute of Technology, Pasadena, California 91125, USA; c Okinawa Institute of Science and Technology Graduate University, Onna, Okinawa 904-0495, Japan

**Keywords:** X-ray magnetic diffraction, resonant X-ray orbital scattering, non-resonant X-ray diffraction of charge orders, high pressure, cryogenic temperatures, spin-density-wave materials, antiferromagnets

## Abstract

Advances in both non-resonant and resonant X-ray magnetic diffraction since the 1980s have provided researchers with a powerful tool for exploring the spin, orbital and ion degrees of freedom in magnetic solids, as well as parsing their interplay. Here, we discuss key issues for performing X-ray magnetic diffraction on single-crystal samples under high pressure (above 40 GPa) and at cryogenic temperatures (4 K). We present case studies of both non-resonant and resonant X-ray magnetic diffraction under pressure for a spin-flip transition in an incommensurate spin-density-wave material and a continuous quantum phase transition of a commensurate all-in–all-out antiferromagnet. Both cases use diamond-anvil-cell technologies at third-generation synchrotron radiation sources. In addition to the exploration of the athermal emergence and evolution of antiferromagnetism discussed here, these techniques can be applied to the study of the pressure evolution of weak charge order such as charge-density waves, antiferro-type orbital order, the charge anisotropic tensor susceptibility and charge superlattices associated with either primary spin order or softened phonons.

## Abstract 2

Resonant and non-resonant X-ray magnetic diffraction under high pressure (above 40 GPa) is comprehensively discussed and reviewed.

## 1. Introduction   

In the early 20th century, quasi-hydro­static pressure in the GPa range was recognized as an elegant and effective tuning technique to continuously manipulate the properties of materials (Bridgman, 1912 ▸). Applying hydro­static pressure provides a means to controllably increase the energy density of a material, broadening bandwidths, increasing the kinetic energy of itinerant electrons and modifying the magnetic exchange interactions between localized spins. In contrast to chemical doping, hydro­static pressure preserves the chemical composition and limits the introduction of disorder in stoichiometric compounds. Unlike a magnetic field, hydro­static pressure does not manifestly break time-reversal symmetry. It is thus not surprising that high-pressure techniques have been employed broadly in fields from condensed-matter physics and materials science to chemistry, geology and planetary science.

In order to substantially change a material’s behavior, the injected energy density typically needs to be of the same order as the electronic energy density, often characterized by the chemical potential or Fermi energy. Pressure as an expression of energy density, with a conversion rate of 1 GPa = 6.3 meV Å^−3^, is thus of great interest for all of the above-mentioned disciplines. While a variety of techniques have been developed to reach these high pressures (Eremets, 1996 ▸), diamond-anvil cells (DACs) have become the most widely used system. Compared with other opposing or cubic anvil types of pressure vessels, DACs offer a combination of advantages in size, cost, accessible pressure range, ease of operation, safety and compatible measurement techniques.

A variety of experimental techniques have been developed to probe both ground states and excitations of ion, charge and spin degrees of freedom in a DAC-based pressure environment. These include electrical transport (Derr *et al.*, 2008 ▸; Jaramillo *et al.*, 2010 ▸), AC magnetic susceptibility (Debessai *et al.*, 2009 ▸; Palmer *et al.*, 2015 ▸) and heat capacity/calorimetry (Demuer *et al.*, 2000 ▸) for studying the bulk properties of materials under pressure. Nuclear magnetic resonance (Eremets, 1996 ▸), Mössbauer spectroscopy (Bi *et al.*, 2016 ▸), X-ray magnetic dichroism (Duman *et al.*, 2005 ▸) and fluorescence (Bi *et al.*, 2016 ▸), as well as optical probes (Eremets, 1996 ▸), allow characterization of local behavior. We focus in this article on high-pressure X-ray magnetic diffraction in a DAC environment, techniques that have emerged over the last two decades. The ability to combine magnetic and charge X-ray diffraction in the same experimental cell paints a more complete picture of the underlying physics in a material’s evolution with pressure. Direct microscopic insight into magnetic order at a pressure-driven quantum phase transition, for example, including both the spin structure and wavevector, can be correlated with the behavior of the lattice and orbital resonances. X-ray diffraction is thus a particularly valuable technique for research on emergent materials, competing states and quantum criticality given the advantages of pressure as an athermal tuning technique for manipulating the quantum nature of charge and spin order.

Neutron diffraction is also a powerful tool for probing charge and spin degrees of freedom. However, X-rays have a number of advantages within the constraints of high-pressure experiments. Neutrons have strong intrinsic absorptions for the naturally abundant isotopes of elements such as Gd, Sm, Eu, B, Cd, Dy and Ir, often requiring the growth of isotopically enriched specimens. Limitations in crystal-growth techniques, quality and cost often make it prohibitively difficult to grow single crystals sufficiently large for neutron diffraction. Similarly, because neutron beams are not easily focused down to a small size, typical neutron-diffraction high-pressure cells are massive, and hence are difficult to efficiently cool and then pressure tune once cold (Mirebeau, 2007 ▸; Klotz, 2013 ▸). Neutron magnetic diffraction on powder samples is typically limited to pressures below 25 GPa even with the strongest Paris–Edinberg cells (Klotz, 2013 ▸), and magnetic single-crystal diffraction is further limited to pressures below 10 GPa (Mignot *et al.*, 2000 ▸; Klotz, 2013 ▸), as the need to maintain a large range of accessible reciprocal space limits the strength of the pressure cell. X-ray magnetic diffraction can overcome many of these challenges and provides a parallel pathway to neutrons for direct insight into magnetism under pressure.

Both non-resonant (Platzman & Tzoar, 1970 ▸; de Bergevin & Brunel, 1981 ▸; Blume, 1985 ▸; Gibbs *et al.*, 1985 ▸; Blume & Gibbs, 1988 ▸; Hill *et al.*, 1995 ▸) and resonant (Gibbs *et al.*, 1988 ▸; Blume, 1994 ▸; Hill & McMorrow, 1996 ▸; Caciuffo *et al.*, 2002 ▸; Paolasini *et al.*, 2007 ▸; Yamaura *et al.*, 2012 ▸; Sagayama *et al.*, 2013 ▸; Strempfer *et al.*, 2013 ▸; Donnerer *et al.*, 2016 ▸) types of X-ray magnetic diffraction were enabled by the introduction of second-generation synchrotron radiation sources in the 1980s, as the high flux density of these sources compensates for the small magnetic cross-sections of both of the X-ray scattering types. Although X-ray magnetic diffraction was proven feasible for ferromagnets (de Bergevin & Brunel, 1981 ▸) and ferrimagnets (Kim *et al.*, 2007 ▸) in both powder (de Bergevin & Brunel, 1981 ▸; Kim *et al.*, 2005 ▸) and single-crystal forms, these techniques are used predominantly for exploring antiferromagnetic single crystals.

X-ray magnetic diffraction at high pressure has become accessible over the past 15 years, with examples of both non-resonant and resonant diffraction. These include studies of antiferromagnetic Cr (Feng *et al.*, 2007 ▸, 2015*a* ▸), CeFe_2_ (Wang *et al.*, 2012 ▸), GdSi (Feng *et al.*, 2014 ▸), MnP (Wang *et al.*, 2016 ▸), CeFe_2_ at the Ce *L*_3_ edge (*E* = 5.720 keV) (Kernavanois *et al.*, 2005 ▸; Braithwaite *et al.*, 2006 ▸; Paolasini *et al.*, 2007 ▸), γ-Li_2_IrO_3_ (albeit without polarization analysis by Breznay *et al.*, 2017 ▸) and Sm_2_Ir_2_O_7_ (Wang *et al.*, 2019 ▸) at the Ir *L*_3_ edge (*E* = 11.214 keV) and Cd_2_Os_2_O_7_ at the Os *L*_2_ edge (*E* = 12.387 keV) (Wang *et al.*, 2018 ▸). Recently, we have extended these techniques to samples pressurized to 40 GPa and beyond at temperatures down to ∼3.5 K. With fourth-generation synchrotron radiation sources on the horizon offering enhanced brilliance and total flux (Hettel, 2014 ▸), we hope that a detailed description of the techniques of high-pressure X-ray magnetic diffraction will promote broader interest and understanding in the community.

## 2. Synergy of X-ray magnetic diffraction, high pressure and cryogenics   

Successful high-pressure magnetic diffraction requires synergy between synchrotron-based X-ray optics, high-pressure instrumentation and cryogen-free cryogenics. To optimize the measurement efficiency under the constraints of a synchrotron radiation source, emphasis needs to be placed on (1) the components of both the X-ray diffraction and the high-pressure setups, as well as (2) maintaining sample quality in a low-temperature, high-pressure environment. Cryogen-free cryogenics are preferred over the traditional liquid-helium-based cryogenics because of the ease of operation, continuous operation at a fixed low temperature through a week-long experiment, and the full rotational freedom to allow access to multiple diffraction orders. General principles of X-ray diffraction from weak charge and magnetic order under high pressure and cryogenic temperature have been discussed previously (Feng *et al.*, 2010 ▸). We describe here the particular demands and challenges of resonant diffraction at relatively low X-ray energies (∼10 keV) and higher pressures (∼40 GPa). The discussion is primarily based on our experience at Sector 4-ID-D of the Advanced Photon Source (APS), but the general principles are applicable to beamlines at many different synchrotron radiation sources (Paolasini *et al.*, 2007 ▸; Strempfer *et al.*, 2013 ▸).

### 2.1. X-ray optics   

**Figure 1:**
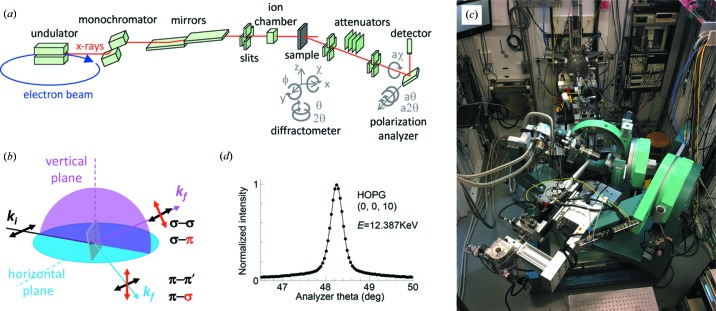
(*a*) Overall layout of optical components of the X-ray magnetic diffraction in the horizontal diffraction geometry; two additional degrees of rotational freedom in the vertical plane are not specified. Note that the extra degree of rotational freedom (ϕ) of the sample controls its azimuthal angle φ. (*b*) Two choices of diffraction geometry. The linearly polarized X-rays from the synchrotron provide either a π (in-plane for horizontal diffraction) or a σ (out-of-plane for vertical diffraction) initial condition. (*c*) Aerial view of the horizontal diffraction experimental setup in the experimental hutch of Sector 4-ID-D of the APS. (*d*) Measured mosaic profile (∼0.35° FWHM) of a 5 mm thick, highly oriented pyrolytic graphite (HOPG) polarization analyzer for X-rays at the Os *L*_2_ resonance edge (12.387 keV).

We provide in Fig. 1 ▸(*a*) a schematic overview of the X-ray setup currently at Sector 4-ID-D of the APS at the Argonne National Laboratory. X-rays are generated by circulating electrons in the synchrotron and are ∼99% polarized in the horizontal plane. The initially broadband X-rays are monochromated by a combination of two nearly perfect single crystals. For a symmetric Si(111) monochromator, the X-ray energy resolution is Δ*E*/*E* ≃ 1.32 × 10^−4^ full width at half-maximum (FWHM), which is the major factor in determining the reciprocal space resolution. Finer resolution is possible at the cost of reductions in the X-ray flux. Utilizing a pair of single crystals brings the monochromatic X-rays to the horizontal direction, improves X-ray energy stability by removing the heat load on the second crystal and allows a detuning process to remove higher harmonics of the primary X-rays.

With current third-generation synchrotron radiation sources, a typical X-ray beam has a cross-section of 2.5 × 1.0 mm and is then focused by metallic (Pd at 4-ID-D) coated mirrors down to approximately 250 × 120 µm. Motorized slits further reduce the beam to a size comparable to the typical lateral sample size in a diamond-anvil cell: 70 × 70 to 100 × 100 µm. Smaller sample and X-ray beam sizes could be used although they need to exceed the lower limit set by cryostat vibrations (∼30 µm in our system). The metallic coating on the mirror should effectively reject higher harmonics. At Sector 4-ID-D of APS, the beam intensity on our high-pressure sample, about 2 × 10^12^ photons s^−1^ at 20 keV and 4.5 × 10^12^ photons s^−1^ at 12.387 keV, is roughly 1/8th that of the unfocused monochromatic X-ray beam emerging from the monochromator.

A Huber 5021 diffractometer with nine circles provides rotational freedom for both the sample and the analyzer, in either the vertical or the horizontal plane (Fig. 1 ▸). As all of the rotation axes of the diffractometer (except the polarization analyzer) meet at the sphere of confusion with a radius of about 50 µm, comparable with both the sample size in a diamond-anvil cell and the focused X-ray beam cross-section (∼100 µm), a set of motorized *x*–*y*–*z* translational stages is necessary to move the sample into the X-ray beam every time a new diffraction order is approached. It is desirable to place the *x*–*y*–*z* stages, such as a Huber 5106.20M, inside the last rotational circle to hold a Gifford–McMahon type cryostat (Sumitomo RDK-205E) with 0.5 W cooling power at *T* = 4 K. We removed the flanges on the first and second cooling stages of this higher-cooling-power model in order to fit it onto the diffractometer sample stage.

In general, there are two diffraction geometries for opposing-anvil pressure vessels. The reflection (Bragg) type use either the side surface of a plate sample through a beryllium gasket close to the anvil culet plane (Kernavanois *et al.*, 2005 ▸) or through the same diamond anvil in a backscattering geometry. The transmission (Laue) type let X-rays go through both the sample body and the anvils (Feng *et al.*, 2010 ▸, 2014 ▸). For the Bragg diffraction geometry through the Be gasket, there is very limited reciprocal space access. As discussed below, the Laue geometry provides much greater freedom in the azimuthal range and a higher level of tolerance for sample misalignment, both during preparation and from motion inside the pressure chamber during pressurization.

To be sensitive to the weak diffraction signals, it is best to use a tight collimation, such as Huber 3002.60M tube slits, to reject random elastic scattering along the incident X-ray beam path, rather than two-dimensional image plate detectors (Feng *et al.*, 2005 ▸). The detector slits are located about 0.85 to 1.0 m away from the sample along the detection 2θ arm and can provide a reciprocal space resolution of ∼1 × 10^−3^ Å^−1^ with a 100 µm sized opening for 20 keV X-rays. It is also best to use single-element X-ray detectors such as NaI-based scintillation detectors (CyberStar, Oxford Danfysik) with a large detection area of tens of mm^2^ or a CMOS-based silicon-drift detector (Vortex, Hitachi) with a high-energy resolution to exclude X-ray fluorescence, in accordance with the constraints of the tight collimation.

Non-resonant diffraction allows one to choose an X-ray energy that is not confined to elemental absorption edges and typically ranges from 10 to 20 keV. This range minimizes absorption by the diamond anvils, is a better match to typical sample thicknesses and could avoid contamination from sample-originated X-ray fluorescence. It also expands the Ewald sphere so that more diffraction orders are within the 2θ angular range of the DAC (70° in our setup). Combined with a suitable mirror, higher energy X-rays can also obviate contamination by higher harmonics (Wang *et al.*, 2012 ▸). However, because of the weak cross-section, non-resonant diffraction is typically restricted to exploring incommensurate spin and charge order (Feng *et al.*, 2007 ▸, 2012 ▸, 2015*b* ▸; Hücker *et al.*, 2010 ▸).

Resonant X-ray diffraction is element specific, and thus can be extremely useful when two species of magnetic ions are present and need to be distinguished. The resonant enhancement for magnetic diffraction is typically a factor of 10–1000, which is offset, at least in part, by the low reflectivity (1–2%) of a typical HOPG polarization analyzer. More profoundly, X-ray polarization analysis, a standard component of resonant magnetic diffraction, brings major new capabilities to the experiment. It allows for separate explorations of the polarization preserving (σ–σ)/(π–π′) and the polarization switching (σ–π)/(π–σ) channels [Fig. 1 ▸(*b*)], with distinctive forms in energy scans across the resonant edge. To perform polarization analysis of X-rays up to ∼13 keV, a 25 × 25 × 5 mm plate of HOPG was used as an analyzer, chosen primarily for its spatial uniformity and relatively broad mosaic profile [0.35° FWHM, Fig. 1 ▸(*d*)]. This mosaic width is a compromise between the analyzer and samples under pressure in order to match the angular reception range between them. Methods for keeping the sample mosaic below 0.5° FWHM will be discussed in the following section.

### 2.2. The high-pressure sample environment   

**Figure 2:**
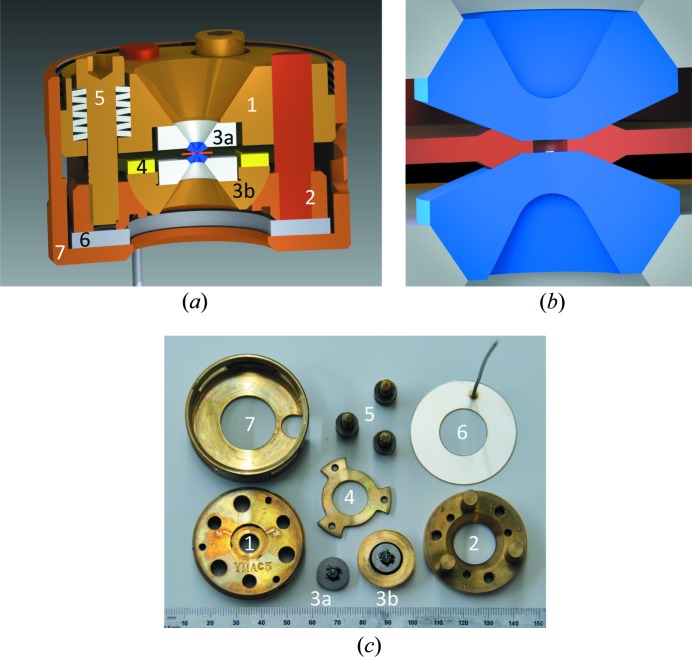
(*a*) Cross-sectional view of a modified three-pin Merrill–Bassett type diamond-anvil pressure cell (Merrill & Bassett, 1974 ▸). (1, 2) Upper and lower pieces of the cell body. Pins press-fit into the lower part align the two pieces with each other. (3*a*, 3*b*) Rear-perforated diamonds mounted on tungsten-carbide seats. One seat is mounted to a rocker for angular alignment. A retaining ring (4) holds the rocker and seat in position. Screws and stacks of Belleville disk washers (5) provide the sealing force and initial room-temperature pressurization. A helium bellows actuator (6) and retaining cap (7) allow for *in situ* cryogenic pressurization. (*b*) Magnified cross-sectional view of the center of the cell showing the wide-angle perforation design based on the Boehler diamond anvil (blue), along with a gasket (orange) and a typical sample (white). (*c*) Photograph of actual cell components with numbers corresponding to those in panel (*a*).

Because of their relatively low mass, diamond-anvil pressure cells are better suited than other types of opposing and cubic anvil cells for incorporation into a cryogenic environment at a synchrotron radiation source. We use cells made from silicon aluminium bronze (C64200) (Fig. 2 ▸), which does not need heat treatment and hence is straightforward to be machined to high precision. Silicon aluminium bronze also has excellent anti-galling and anti-seizing properties under non-lubricated and vacuum conditions. The original Merrill–Bassett design (Merrill & Bassett, 1974 ▸) was modified to allow pins with a larger diameter (1/4 of an inch) for better stability (Fig. 2 ▸). To improve efficiency, pressure was varied *in situ* using a helium diaphragm or membrane [Daniels & Ryschkewitsch, 1983 ▸; Sinogeikin *et al.*, 2015 ▸ and see Fig. 2 ▸(*c*)], removing the need to thermally cycle to room temperature for each pressure change.

The high-pressure environment always increases the elastic scattering background, with contributions from both the anvil and the pressure medium, as well as random artifacts such as sharp diffraction dips (Loveday *et al.*, 1990 ▸). These issues are alleviated by several developments in anvil design that provide a large reciprocal space access with a 2θ range of 70° (Boehler & Hantsetters, 2004 ▸). Thinning the rear of both anvils with wide-angle conical (2θ = 60°) perforations [Fig. 2 ▸(*b*)] provides a further reduction in the total diamond thickness in the X-ray path to 0.9–1.0 mm (Feng *et al.*, 2014 ▸, 2015*a* ▸; Wang *et al.*, 2016 ▸, 2018 ▸).

Even with these improvements in reducing the background, the success of X-ray magnetic diffraction under high pressure heavily relies on the quality of single-crystal samples. It is critical to preserve the initial crystal quality through the entire cooling and pressurization process. Many examinations of high-pressure hydro­staticity focused on the static condition at a fixed pressure (Klotz *et al.*, 2009 ▸). With single-crystal samples of DAC-compatible size properly prepared for loading (Feng *et al.*, 2005 ▸; Rivers *et al.*, 2008 ▸), preserving sample quality during pressurization requires considering both the construction of the initial pressure chamber and the relaxation of the pressure medium during the pressurization process. As we noted previously (Feng *et al.*, 2010 ▸), the choice of pressure medium can help maintain a large chamber-to-sample volume ratio as the pressure increases. A typical pressure chamber has an initial volume of order 0.02 mm^3^ and a chamber-to-sample ratio of about 100:1. Some highly compressible noble gases, such as helium and neon, which are typically loaded in a supercritical state of 0.1–0.2 GPa at *T* = 300 K (Rivers *et al.*, 2008 ▸; Klotz *et al.*, 2009 ▸; Feng *et al.*, 2010 ▸), would significantly reduce their own volumes to 5–10% of the initial volume at high pressure and low temperature, and thus require a very small and thin sample (such as 10 × 10 × 5 µm by Rivers *et al.*, 2008 ▸). Such small samples cannot take advantage of the full flux of a focused synchrotron X-ray beam. We regard a methanol:ethanol 4:1 mixture as an ideal pressure medium up to at least 40 GPa, combining both quasi-hydro­staticity and low compressibility to preserve the quality of single-crystal samples in a voluminous pressure chamber. A set of the best-condition sample-chamber parameters are listed in Table 1 ▸ based on our experience, which allow reaching pressures comparable to the estimated limits given by Dunstan & Spain (1989 ▸) while minimizing pressure anisotropy.

Pressurization is a mechanical process that is always non-adiabatic. For opposing-anvil devices, the evolution of a sample chamber’s lateral diameter and vertical thickness is well discussed in the literature (Eremets, 1996 ▸). In addition, diamond anvils tend to elastically buckle towards the center under pressure (Eremets, 1996 ▸). These shape changes during pressurization lead to the establishment of new pressure gradients in both the gasket and the pressure medium, followed by a slow relaxation towards a quasi-hydro­static condition, with the ultimate hydro­staticity limited by the shear modulus of the medium and the relaxation rate determined by the geometry of the pressure chamber. This redistribution of the pressure medium through plastic deformation and rheology inside a shape-changing chamber is a main characteristic of diamond-anvil cells. It suggests that a voluminous pressure chamber is always preferable as such a chamber effectively reduces the damage from large movements of the pressure medium on the sample. This is desirable as the strains from such motion can degrade the sample surface, impacting the quality of reflection­-geometry-based measurements such as Bragg diffraction and optical Raman. The pressure-medium relaxation process is a function of both the temperature and the pressure scale, and at *T* ≃ 4 K relaxation times can be in the order of 30 min below 10 GPa and up to several hours at 30–40 GPa.

**Figure 3:**
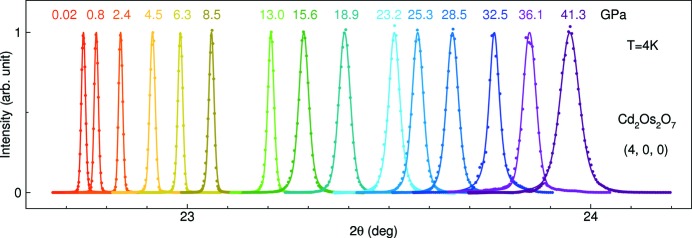
Longitudinal (θ–2θ) diffraction line shapes of the (4, 0, 0) lattice order of Cd_2_Os_2_O_7_. The measurements were carried out using 12.387 keV X-rays in a Laue (transmission) geometry through samples of ∼15 µm thickness. The system remains in the cubic structure up to 41 GPa, while the gradually broadened line shapes with increasing pressure demonstrate the pressure-gradient condition experienced by the single-crystal sample.

Our constructed sample pressure environment can be evaluated sensitively by the lattice diffraction line profiles of single-crystal samples. In Fig. 3 ▸, we exemplify the level of pressure homogeneity by plotting diffraction line profiles of the (4, 0, 0) order in Cd_2_Os_2_O_7_ from 0 to 41 GPa. As expected, the line shape broadens as a reflection of the increasing pressure gradient in the sample chamber. Nevertheless, the diffraction lines all remain a single peak profile with symmetric shapes. From 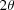 diffraction line profiles in Fig. 3 ▸, the pressure gradient can be estimated as 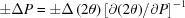, where 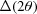 is the half width at half-maximum of the diffraction profile and 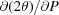 is the linear lattice compressibility measured by 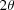. The gradient at 41 GPa is estimated to be the highest at ±1.4 GPa, is reduced to ±0.7 GPa at 30 GPa and is reduced to ±0.1 GPa below 10 GPa. Alternatively, the FWHM of the diffraction lines indicate a lattice coherence length evolving from resolution-limited (>1700 Å) at 0 GPa to ∼350 Å at 41 GPa. Similar diffraction line profile evolutions of (1, 1, 1) and (2, 2, 0) are shown in the work by Wang *et al.* (2018 ▸). These single-crystal diffraction line profiles provide a critical evaluation of the sample pressure condition and are much more sensitive than other evaluation methods using polycrystalline materials, such as ruby line shapes and Ag diffraction profiles (Feng *et al.*, 2010 ▸). Our pressure condition, set by a combination of sample-chamber construction in Table 1 ▸ and pressure-medium choice, meets the desired stringent sample-quality requirement necessary to perform X-ray magnetic diffraction.

In general, the sample thickness should be kept at no more than one absorption length for X-rays of the specific energy, but also should be much less than the high-pressure chamber height. For resonant studies, the absorption-length condition is typically the more restrictive one; for example, the resonant-diffraction study at the Os *L*_2_ edge (*E* = 12.387 keV) of Cd_2_Os_2_O_7_ described below requires the sample to be polished down to a thickness of 13–15 µm.

## 3. Non-resonant X-ray diffraction of incommensurate spin and charge order   

The instrumentation we outlined above can be applied widely to both non-resonant and resonant X-ray diffraction under high pressure. Here we discuss the case of non-resonant diffraction, with an emphasis on exploring incommensurate antiferromagnetic order and charge order, including charge-density waves, and charge superlattices of either magnetic order or softened phonons (Feng *et al.*, 2007 ▸, 2011 ▸, 2012 ▸, 2014 ▸, 2015*b* ▸; Wang *et al.*, 2012 ▸; Hücker *et al.*, 2010 ▸; Wang *et al.*, 2016 ▸). In the next section, we will demonstrate resonant diffraction of commensurate antiferromagnetic order, which can be extended to charge-based resonant scattering of anisotropic tensor susceptibility (ATS) and orbital order under pressure.

Non-resonant X-ray magnetic scattering is purely a relativistic effect of quantum mechanics and is non-existent in its non-relativistic limit (Platzman & Tzoar, 1970 ▸). Nevertheless, the scattered radiation can be qualitatively understood as arising from magnetic dipoles of electrons, by analogy to Thompson scattering of electric dipoles (Platzman & Tzoar, 1970 ▸; de Bergevin & Brunel, 1981 ▸). The ratio of scattering amplitudes between these two types of dipoles is essentially a ratio between the Compton wavelength 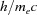 of the electron and the radiation wavelength 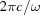 (Platzman & Tzoar, 1970 ▸), or equivalently between the radiated X-ray energy 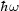 and the electron-rest-mass energy 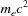. The cross-section of non-resonant X-ray magnetic scattering, as the square of the scattering amplitude, is thus smaller by 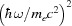 to that of the charge scattering (de Bergevin & Brunel, 1981 ▸; Blume, 1985 ▸; Blume & Gibbs, 1988 ▸).

At intermediate X-ray energies (*E* < 10 keV), this non-resonant technique is capable of separating the spin and orbital contributions to the magnetic structure using X-ray polarization analysis (Gibbs *et al.*, 1988 ▸; Caciuffo *et al.*, 2002 ▸). However, not only is such an exploration time consuming, but a detailed analysis of this cross-section [equations (6) and (8) of Blume & Gibbs, 1988 ▸] also indicates that for hard X-rays (*E* > 10 keV) the non-resonant magnetic cross-section is increasingly dominated by either σ–σ or π–π′ matrix elements [with a leading order of ∼sin^2^(θ)] at low angles, without significant X-ray polarization flipping or orbital components [with a leading order of ∼sin^4^(θ)]. The cross-section is vanishingly small at large 2θ because of the fast drop-off of the magnetic form factor at large transferred momentum **q** (Blume, 1985 ▸). Hence, non-resonant magnetic diffraction in the hard X-ray regime (such as *E* = 20 keV) is difficult to distinguish from charge diffraction *per se* and is mostly sensitive to the component of the staggered spin moment that is perpendicular to the diffraction plane, spanned by the incoming and scattered wavevectors, **k** *_i_* and **k** *_f_*, respectively [Fig. 1 ▸(*b*)]. However, the cross-section of non-resonant magnetic scattering can be calculated quantitatively, leading to an experimental determination of the staggered moment size (Hill *et al.*, 1995 ▸; Wang *et al.*, 2012 ▸; Feng *et al.*, 2007 ▸; Wang *et al.*, 2016 ▸).

**Figure 4:**
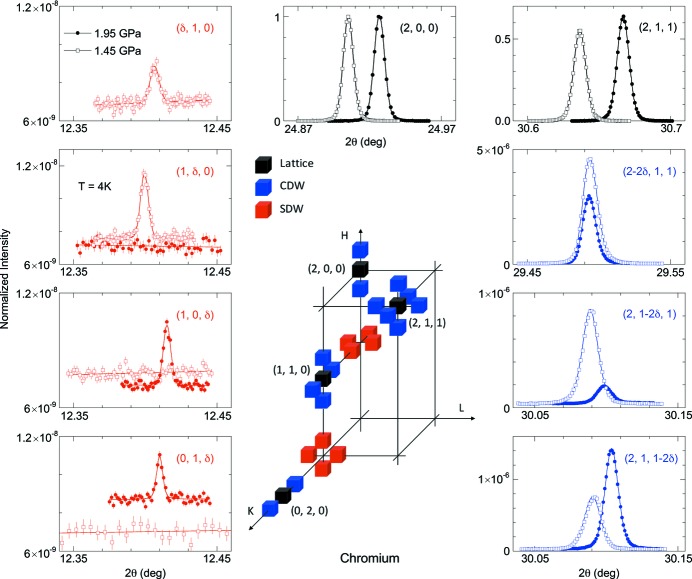
Longitudinal (θ–2θ) line shapes of both magnetic and charge-diffraction patterns of the lattice (black), CDW (blue) and SDW (red) orders in Cr under pressure, using non-resonant X-ray techniques, adapted from the work by Feng *et al.* (2015*a* ▸). The schematic drawing represents locations of diffraction orders in reciprocal space. The sample was prepared in a plate shape with a surface normal along the *L* direction (0, 0, 1). While most diffraction patterns of interest are within or close to the *H*–*K* zone, CDW states are best probed with non-vanishing cross-section for all three domains around (2, 1, 1), which is accessible within our high-pressure cell’s construction (a full 70° 2θ range from both sides, Fig. 2 ▸). For each pressure, all diffraction intensities are normalized to that of the (2, 0, 0) order. The two (2, 0, 0) order intensities at 1.45 and 1.95 GPa are consistent within 6%. The contrast between X-ray magnetic diffraction data from two pressures indicate a spin-flip transition of the SDW state in Cr from longitudinal to transverse relative to the propagating ***Q*** vector. The null intensity of the (δ, 1, 0) order at 1.95 GPa was not measured because of expiring beam time.

Non-resonant diffraction of weak incommensurate antiferromagnetic order under pressure was first demonstrated in Cr in the work by Feng *et al.* (2007 ▸), with the general experimental setup discussed by Feng *et al.* (2010 ▸). The major improvement over the last decade has been the installation of wide conically perforated diamond anvils (Feng *et al.*, 2014 ▸) (Fig. 2 ▸) and a better understanding of the pressure-chamber construction (Table 1 ▸). The improved efficiency with these updated design parameters can be observed through a recent non-resonant diffraction study of both the spin-density wave (SDW) and charge-density wave (CDW) states in Cr across its pressure-induced spin-flip transition, as the data quality in the work by Feng *et al.* (2015*a* ▸) and Fig. 4 ▸ is significantly improved by comparison with the early work by Feng *et al.* (2007 ▸).

With the limited range of accessible reciprocal space inherent in high-pressure measurements, it is important to prepare samples in a proper geometry with the constraints of both the high-pressure cell and diffraction cross-section in mind. As an example, we consider the case of probing the SDW and CDW states in Cr (Fawcett, 1988 ▸). At ambient pressure, the general X-ray diffraction pattern and cross-sections of CDWs and SDWs in Cr are well documented (Hill *et al.*, 1995 ▸). Because of the cubic symmetry of Cr in the paramagnetic phase, there is a macroscopic degeneracy of three CDW domains forming along either the *H*, *K*, or *L* directions below *T*_N_ = 311.5 K. Within each CDW domain, there exists either one SDW domain with a longitudinal spin structure and moments parallel to the ordering wavevector **Q** = (1−δ, 0, 0) ≃ (0.95, 0, 0), or two degenerate SDW domains with a transverse spin structure and moments perpendicular to **Q**. The CDW, with wavevectors such as (2δ, 0, 0) and (0, 2δ, 0), is considered the second harmonic of the SDW (Fawcett, 1988 ▸). Because of their differing wavevectors, the CDW and SDW diffraction patterns are observed at different reciprocal space positions (Fig. 4 ▸). In comparison with the (2, 0, 0) order, CDWs have a cross-section of 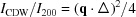, with **q** the scattering wavevector and Δ the longitudinal CDW displacement amplitude (Hill *et al.*, 1995 ▸), which is much larger than that of the SDW. Thus, the CDW diffraction intensities can be used to calibrate the relative volumes of the three (*H*, *K*, *L*) SDW domains.

In order to explore the pressure-induced spin-flip transition, plate-shaped Cr samples were prepared with a surface normal of (0, 0, 1). Taking into account the sensitivity of non-resonant diffraction to spin components perpendicular to the diffraction plane, two sets of patterns, (1, δ, 0) and (δ, 1, 0) versus (1, 0, δ) and (0, 1, δ) for longitudinal and transverse spin structures, respectively, would become observable in each phase, while the other set becomes simultaneously distinct (Hill *et al.*, 1995 ▸; Feng *et al.*, 2015*a* ▸). Fig. 4 ▸ illustrates this contrast in diffraction patterns between these two types of SDW order in the longitudinal and transverse phases at 1.45 GPa and 1.95 GPa, respectively. The change of diffraction pattern is seen clearly, despite the low intensity of the magnetic diffraction signal, of order 10^−9^ times the peak intensity of the main (2, 0, 0) order.

With a surface normal vector (0, 0, 1), the sample *H*-*K* plane is aligned in parallel to the anvil culet plane and is typically the most accessible part of the reciprocal space. However, CDW satellites around orders such as (2, 0, 0), (1, 1, 0) and (0, 2, 0), are sufficiently close to the *H*-*K* plane that the *L*-axis CDW domain is not optimized for probing by X-ray diffraction, as the scattering cross-section progresses as ∼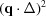 (Hill *et al.*, 1995 ▸). Therefore, it is necessary to reach a diffraction order where *H*, *K* and *L* are all non-zero, such as (2, 1, 1). For a study focusing entirely on the CDW states, samples with a surface normal of (0, 1, −1) were prepared, placing the (2, 1, 1) order within the culet plane (Jaramillo *et al.*, 2009 ▸). For samples with a (0, 0, 1) surface normal, the (2, 1, 1) order is significantly tilted out of the culet plane; the large 2θ = 70° conical solid angle afforded by Boehler anvils (Boehler & Hantsetters, 2004 ▸) allows observation of all three pairs of CDW satellite peaks around the (2, 1, 1) order at reasonably high levels of intensity, using 20 keV X-rays in the non-resonant condition (Fig. 4 ▸). With this approach, we are able to simultaneously track lattice, CDW, and SDW degrees of freedom at every pressure across the spin-flip transition in Cr.

As noted above, the scattering cross-section for polarization switching in non-resonant diffraction is mostly undetectable. This makes it imperative to be able to distinguish the magnetic diffraction signal from spurious charge-scattering signals. Although it is natural to consider a temperature-evolution study, the very low counting statistics of typical non-resonant magnetic diffraction signals, combined with the limited time of a synchrotron experiment, argue against such an approach. Unlike resonant scattering (see below), the most time-consuming part of non-resonant diffraction is to accumulate the statistics of a few reciprocal space scans (either θ/χ/θ–2θ or *H*/*K*/*L* types), with each easily requiring four to six hours. Instead, it is more efficient to explore magnetic diffraction patterns at one (*P*, *T*) point in order to verify their magnetic nature. For example, in Cr, CeFe_2_, GdSi and MnP, the single-crystal nature of the magnetic peak was first verified by three independent reciprocal space scans [*e.g*. θ/χ/θ–2θ scans in the work by Wang *et al.* (2016 ▸)]. In each of these cases, at least one pair of magnetic diffraction peaks were identified that possess a mirror symmetry relative to the reciprocal lattice structure (Fig. 4 ▸ and in the works by Feng *et al.*, 2007 ▸, 2014 ▸, 2015*a* ▸; Wang *et al.*, 2012 ▸; Wang *et al.*, 2016 ▸). In the cases of Cr, GdSi and MnP, the pair of magnetic satellites were also tracked relative to the reciprocal lattice through several different pressures (Feng *et al.*, 2015*a* ▸; Wang *et al.*, 2016 ▸). Not only was the diffraction intensity verified with the non-resonant X-ray magnetic diffraction cross-section for extraction of effective moment sizes in each system studied (Cr, GdSi and MnP) but it is also possible to measure multiple reciprocal space points, such as nine orders (including three null) in CeFe_2_ at *P* = 3.3 GPa and *T* = 3.5 K, so the magnetic intensities could be used to check consistency with models of non-coplanar spin structure (Wang *et al.*, 2012 ▸). A fully *ab initio* refinement of spin structure, requiring measuring intensities at tens of magnetic diffraction orders is unfortunately not likely; before the availability of the wide-perforation diamond anvils, the measurement of all magnetic and lattice information at that single (*P*, *T*) point of CeFe_2_ consumed an entire six day allocation of beam time.

## 4. Resonant X-ray diffraction of commensurate spin, charge and orbital order   

Since the first demonstration of resonant X-ray magnetic diffraction three decades ago (Gibbs *et al.*, 1988 ▸), this technique has illustrated numerous antiferromagnetic structures at ambient pressure, where neutron magnetic diffraction was difficult because of either strong neutron absorption without isotope enrichment or small single-crystal size. The all-in–all-out (AIAO) type of spin order in Os- or Ir-based pyrochlore compounds (such as Cd_2_Os_2_O_7_ and *R*_2_Ir_2_O_7_ with *R* = Eu, Sm and Nd) embodies both difficulties and represents by far one of the most sophisticated spin structures that are studied predominantly by resonant X-ray magnetic diffraction techniques (Yamaura *et al.*, 2012 ▸; Sagayama *et al.*, 2013 ▸; Donnerer *et al.*, 2016 ▸; Wang *et al.*, 2018 ▸). Residing locally on the atomic sites of a pyrochlore lattice, the AIAO spin order has all four spins at the corner of each tetrahedron pointing along the local 〈1, 1, 1〉 axis either all towards the tetrahedron center or away from it, creating a highly symmetric three-dimensional spin structure that is fully compatible with the space group of the underlying pyrochlore lattice (Bramwell & Harris, 1998 ▸). Here, we illustrate some general experimental considerations involved in extending resonant X-ray magnetic diffraction studies to high-pressure environments, using the AIAO order in Cd_2_Os_2_O_7_ as our example.

The continuous tunable energy of synchrotron radiation makes resonant scattering technically feasible by accessing a material’s anomalous absorption and dispersion behavior at characteristic X-ray absorption edges (Blume, 1985 ▸, 1994 ▸). This dispersion anomaly is the origin of multipole features of the scattering cross-section, resonance enhancement, and polarization dependence for both magnetic and charge order. Although the intermediate resonant levels are expected to be empty (Blume, 1994 ▸), the resonant interaction with the excited state is a virtual process, which takes no real time. The description of the resonant process invokes a similarity to the virtually excited photoemission electrons and their multiple scattering off neighboring atoms in the theoretical framework of X-ray absorption near-edge spectroscopy (Dmitrienko *et al.*, 2005 ▸). The diffraction process is elastic with phase coherence preserved across many lattice sites and the resonant description simply reflects the fact that the relativistic nature of the quantum mechanics was simplified to the nonrelativistic framework with a superficial sacrifice of causality.

Resonant virtual transitions between the initial/final state and the intermediate states are categorized into electric dipole or quadrupole types, with higher multipoles possible. This simply reflects a change in quantum number Δ*l* = 1, 2, …, during the resonance, following from a power expansion in (**k·r**) of the transition matrix element exp(*i* **k·r**), with initial and intermediate states specified by *s*, *p*, *d* or *f* types of symmetry (Blume, 1994 ▸). The resonant transition matrix is sensitive to momentum densities of ATS, orbitals and spins (Blume, 1994 ▸; Dmitrienko *et al.*, 2005 ▸). Each quantity then becomes accessible to measurement, although there always can be experimental challenges in separating the effects, as will be illustrated in our discussion of AIAO order below. Electrical dipole transitions typically dominate the resonant scattering cross-section over those of electrical quadrupole and magnetic dipole types (Blume, 1994 ▸; Hill & McMorrow, 1996 ▸). The magnetic dipole type of resonant scattering cross-section is smaller by a factor of 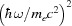 relative to those of the electric dipole type (Hill & McMorrow, 1996 ▸), as described similarly in Section 3 for non-resonant magnetic scattering. Detailed expressions of the magnetic resonant scattering cross-sections are generally complicated but can be found in the literature [*e.g*. Hill & McMorrow (1996 ▸), with equation (15) describing the electric dipole type of resonance].

**Figure 5:**
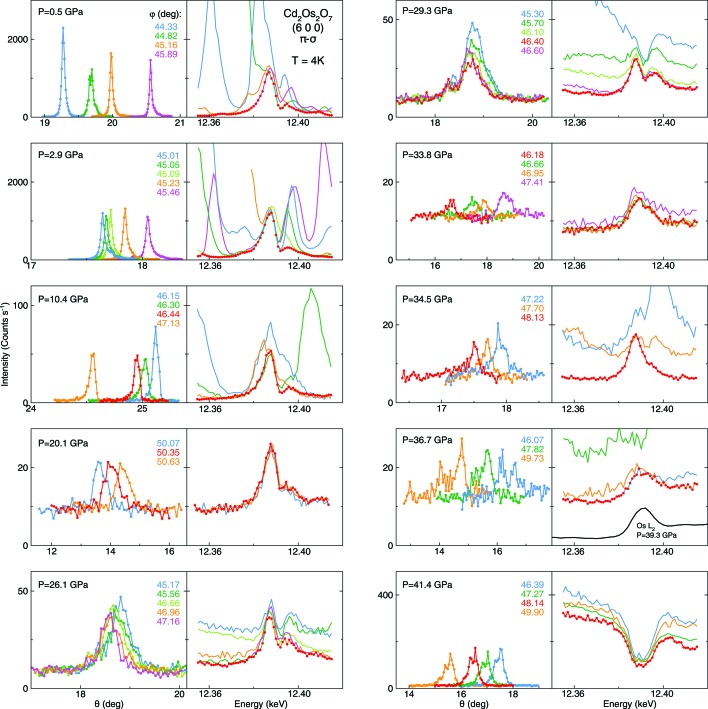
Representative raw resonant X-ray magnetic scattering results on Cd_2_Os_2_O_7_ at ten different pressures, out of 30 total measured pressure points at *T* = 4 K in the work by Wang *et al.* (2018 ▸). The displayed raw data cover six out of the ten highest pressure points explored above 25 GPa, spanning the magnetic quantum phase transition at *P*_c_ = 35.8 GPa. The panels demonstrate in parallel both the sample-mosaic profiles (at *E* = 12.387 keV) and the energy scans at different azimuthal angle φ around 45° relative to the (0, 0, 1) azimuthal vector. At each pressure, we typically study 5 to 12 different azimuthal angle positions, depending on the level of multiple scattering and the convergence of all resonant spectra. The presentation here is limited to three to five sets of azimuthal positions for the sake of clarity, leaving out scans at azimuthal positions which show significant multiple-scattering contamination. The collective set of energy scans is used to determine both the presence and true intensity of the magnetic diffraction, using the lowest common spectral weight designated by red points of either one spectrum or a combination of several spectra. The magnetic diffraction intensity was integrated from sample-mosaic rocking curve(s) of the cleanest energy-scan curve(s) at the resonance energy *E* = 12.387 keV. This allows one to both remove a θ-independent sample fluorescence and minimize the multiple-scattering contamination. At 36.7 GPa, above *P*_c_ = 35.8 GPa, the minimal spectrum of the energy scan differs in shape from a resonance profile. Instead it is similar to the shape of the *L*_2_ absorption edge, indicating that the rounded shape of the energy scan at 36.7 GPa is caused by Os fluorescence. Although all magnetic diffraction intensities at the (6, 0, 0) order are eventually normalized by the (4, 0, 0) lattice diffraction intensity in the π–π′ channel, in order to correct for both the sample size and mosaic difference, our sample mosaic is kept at or below 0.5° FWHM to efficiently match with the polarization analyzer’s mosaic width. For clarity, only the results of the π–σ polarization switching channel are shown here; diffraction results from the π–π′ channel can be found in the work by Wang *et al.* (2018 ▸).

Measured at a fixed finite wavevector, resonant X-ray diffraction is a unique type of spectroscopy, which is typically explored as a function of three major variables: (1) X-ray polarization in and out of the diffraction plane, (2) X-ray energy across the elemental resonance edge, and (3) azimuthal angle about the diffraction order. There are several major physical processes that compete with and potentially obscure a resonantly diffracted magnetic X-ray signal: (1) multiple scattering, (2) ATS scattering and (3) X-ray fluorescence at the resonance edge. The multiple scattering and X-ray fluorescence issues are both rather technical and we reserve their detailed discussion to the caption for Fig. 5 ▸, in close relation to the raw data. Here, we simply note that energy scans across the resonance edge reveal not only the true magnetic spectral weight against multiple scattering but also the nature of the resonant enhancement. While electric dipole transitions typically have resonance energies near the absorption edge of interest, the electric quadrupole transitions are usually found well below the absorption edge (Gibbs *et al.*, 1988 ▸; Caciuffo *et al.*, 2002 ▸).

A major task in performing resonant diffraction of a commensurate spin order consists of a careful evaluation of parameters to resolve the magnetic structure. These include the diffraction geometry, polarization cross-sections, azi­muthal condition, charge ATS form factor, spin structure models and magnetic diffraction order. For the AIAO type of antiferromagnetic order (**Q** = 0) in a pyrochlore lattice, the diffraction intensity can be measured at (*H*, 0, 0) orders, with *H* = 4*n* + 2 positions forbidden for the point-like ionic structure but permitted for both of the resonant types of magnetic and ATS scattering. The polarization-dependent form factors *F* of both types of scattering are specified in the polarization basis of (σ, π) as
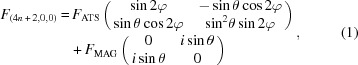
with 2θ the X-ray diffraction angle and φ the azimuthal angle relative to the (0, 0, 1) wavevector [Yamaura *et al.*, 2012 ▸; equation (7) in the supplementary material of Donnerer *et al.*, 2016 ▸]. Thus, the magnetic order has to be probed in the polarization switching channels of either σ–π or π–σ.

Although the magnetic diffraction cross-section does not have an azimuthal dependence, a consequence of the cubic symmetry of the AIAO spin structure, the polarization switching cross-section for ATS scattering can be reduced to zero by choosing a diffraction geometry with an azimuthal angle of around φ = 45° (Yamaura *et al.*, 2012 ▸; Donnerer *et al.*, 2016 ▸). However, the charge-based ATS resonant scattering in the σ–σ and π–π′ channels is maximized at this azimuthal condition, as 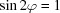. ATS scattering intensities between these two channels differ by a large factor of 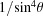, which is ∼110× for the (6, 0, 0) diffraction order of Cd_2_Os_2_O_7_ at the Os *L*_2_ resonant edge (*E* = 12.387 keV). For the (6, 0, 0) diffraction order of Sm_2_Ir_2_O_7_, 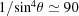 at the Ir *L*_3_ edge (*E* = 11.215 keV). Experimentally, the choice of polarization analyzer is not always ideal at each resonant energy. For example, at the Os *L*_2_ edge, for a HOPG analyzer [with (0, 0, 10) diffraction order, Fig. 1 ▸(*d*)], there is about 1.3% leakage of counts from the π–π′ (σ–σ) channel to the π–σ (σ–π) channel (Wang *et al.*, 2018 ▸). In light of this leakage, it is preferable to choose the diffraction geometry of π–π′, instead of σ–σ, in order to minimize the influence of the ATS resonance signal across the two polarization-analysis channels. Although there are often suitable analyzers such as Au [3, 3, 3] at the Ir *L*_3_ edge (Donnerer *et al.*, 2016 ▸), HOPG is the preferred choice of analyzer for X-ray polarization analysis under high pressure as it allows efficient matching of the angular acceptance range of the analyzer to the sample mosaic, typically controlled under 0.5° FWHM (Figs. 5 ▸ and 6 ▸). With this constraint on the choice of polarization analyzer for high-pressure work, it is of utmost importance to carefully plan the diffraction-geometry condition between π–σ and σ–π in order to maximize the detection efficiency of the magnetic signal.

Without a full refinement, it is beneficial to measure intensities of multiple magnetic orders in order to verify the consistency with a specific spin structure model (Wang *et al.*, 2012 ▸; Donnerer *et al.*, 2016 ▸). To further confirm an AIAO spin configuration, resonant diffraction studies at several (4*n* + 2, 0, 0) orders such as (2, 0, 0), (6, 0, 0), (10, 0, 0) and (14, 0, 0) were performed along with an azimuthal dependence study (Donnerer *et al.*, 2016 ▸). Our work did verify the increasing cross-section of the (6, 0, 0) order relative to that of the (2, 0, 0) order for Cd_2_Os_2_O_7_ under pressure. However, because of the confined angular range of a high-pressure cell, X-ray diffraction of the (10, 0, 0) order is essentially out of the perforated angular acceptance range with an expected 2θ ≃ 62°. Furthermore, at the (6, 0, 0) order our pressure cell can provide an azimuthal range of at least 10° total in combination with a large sample θ range (Fig. 5 ▸). It is unfortunately infeasible to perform a full azimuthal study, such as the example of Sm_2_Ir_2_O_7_ in the work by Donnerer *et al.* (2016 ▸), in a pressure cell and under a Laue diffraction geometry.

Ultimately, the magnetic nature of an X-ray resonance diffraction signal can be verified by its temperature dependence, bounded by the magnetic phase boundary in the (*P*, *T*) phase diagram. Studying the temperature dependence of resonantly scattered intensity tends to be less time-consuming than measuring a series of diffraction patterns, as suggested in the discussion of non-resonant scattering. Under pressurization, a sample’s mosaic typically evolves and hence changes the multiple-scattering condition. A major part of the resonant­-measurement effort at each reciprocal space point thus involves energy scans at many different azimuthal positions in order to extract the common spectral weight of the magnetic resonance behavior (Fig. 5 ▸). On the other hand, a temperature ramp typically does not affect multiple scattering, so long as the pressure stays constant. Once an ideal azimuthal position is identified, the resonant profile is unlikely to be contaminated by extra multiple scattering as temperature changes. We note that while each mosaic or energy scan is relatively efficient (20 min to 1 h), a full temperature evolution study at one pressure can be very time consuming requiring a significant fraction of a typical six day synchrotron-beam allocation. Therefore, we typically only perform such temperature studies very close to the quantum critical point, where the magnetic transition temperature under pressure is close to the measurement base temperature (Wang *et al.*, 2019 ▸), in order to reduce the number of temperature points. Moreover, as pressure vessels are mechanical devices and thermal responses of different cell components vary, there is the possibility for a significant pressure change in response to a large temperature rise (*e.g. T* > 50 K), altering the multiple-scattering condition. The chances of such a pressure variation can be reduced by temperature cycling the pressure cell in order to release the internal mechanical stresses and achieve pressure stability prior to carrying out a detailed temperature study.

## 5. Outlook   

Recent experimental efforts on high-pressure resonant X-ray magnetic diffraction have mainly focused on 5*d* elements such as Ir and Os, with *L* edges in the 11–13 keV range (Breznay *et al.*, 2017 ▸; Wang *et al.*, 2018 ▸; Wang *et al.*, 2019 ▸). Compounds containing 5*d* elements are a vibrant arena for studying intermediately to strongly coupled correlated states, with competing energy scales of the on-site Coulomb interaction, crystal-field effects, spin-orbit coupling and hopping integrals, all well within the 0.1–2 eV range. High-pressure resonant X-ray magnetic diffraction is likely to be extended to the *L* edges of other 5*d* elements such as Hf and Pt in the near future.

By contrast, the *L* edges of 4*f* elements have energies in the 4–10 keV range; these lower energies make resonance studies under high pressure more difficult. Furthermore, magnetic resonant scattering at *L* edges of 4*f* elements relies on either dipole transitions from 2*p* to 5*d* states that are under the influence of the magnetic 4*f* bands or quadrupole transitions from 2*p* to 4*f* bands (Gibbs *et al.*, 1988 ▸). Both processes have much smaller amplitude signals compared with the magnetic resonance at *L* edges of 5*d* elements with direct 2*p* to 5*d* dipole transitions. Indeed, the resonance enhancement factor of ∼50 for Ho (Gibbs *et al.*, 1988 ▸) is respectable but only marginally compensates for the low reflectivity (1–2%) of the polarization analyzer. Given the typically large magnetic moments of 4*f* elements, non-resonant X-ray magnetic scattering could be an alternative approach to probe magnetism of 4*f* compounds under pressure (Feng *et al.*, 2014 ▸). It is intriguing that 3*d* to 5*f* dipole resonances at *M* edges of 5*f* magnets, such as uranium pnictide UAs, provide a much larger resonant enhancement than 2*p*–5*d* dipole resonance at *L* edges of 5*d* elements, mostly because of the larger overlap of the initial and final states (Blume, 1994 ▸). Unfortunately, the *M*-edge resonance X-ray energies of uranium are too low to be easily compatible with a high-pressure setup.

For 3*d* elements, while *L*-edge resonant diffraction in the soft X-ray range is widely employed to probe magnetic and orbital order, *e.g*. manganites and cuprates at Mn and Cu *L* edges (*E* = 639 eV and 930 eV, respectively), there is no significant magnetic resonant enhancement at the *K* edge, *e.g*. the Cu *K* edge in the work by Hill *et al.* (2000 ▸). Again, this is because the dominant dipole 1*s*–2*p* resonance associated with the *K* edge lacks sensitivity to the *d* orbitals that host magnetic spins, which thus precludes convenient studies of 3*d* magnetism with the hard X-ray *K*-edge resonant diffraction needed for compatibility with high-pressure environments. Instead, non-resonant X-ray magnetic diffraction is often the method of choice to reveal magnetic order under pressure in 3*d* compounds such as Cr, CeFe_2_ and MnP (Feng *et al.*, 2007 ▸, 2015*a* ▸; Wang *et al.*, 2012 ▸; Wang *et al.*, 2016 ▸). Similarly, there is no foreseeable possibility to perform resonant X-ray magnetic diffraction in a DAC for 4*d* compounds where the *L* edges are in the energy range of 2–4 keV, so it is necessary to resort to non-resonant magnetic diffraction techniques for pressure measurements.

**Figure 6:**
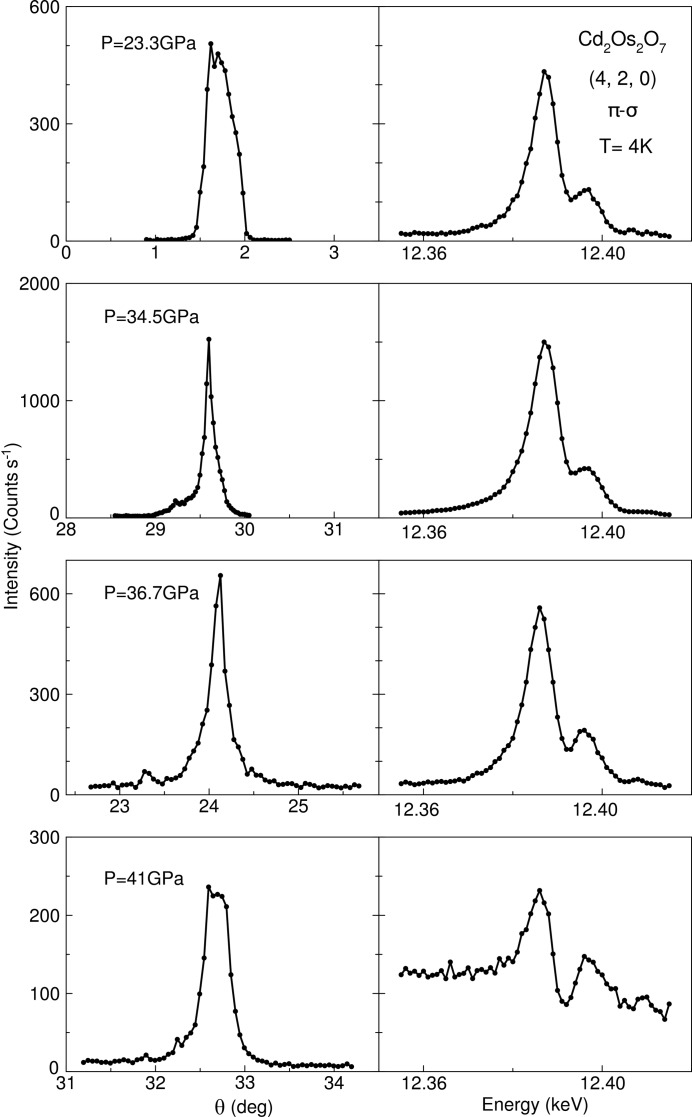
ATS scattering of the charge resonance at the (4, 2, 0) diffraction order in Cd_2_Os_2_O_7_. Both the sample mosaic at *E* = 12.387 keV and energy scans across the Os *L*_2_ edge are compared side-by-side for four different pressures across the pressure-induced magnetic phase boundary at *P*_c_ = 35.8 GPa (Wang *et al.*, 2018 ▸). The high-pressure phase beyond *P*_c_ can be ascribed to the 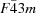 space group, where the lattice (4, 2, 0) order becomes no longer forbidden. The ATS resonance signal is eventually overwhelmed by non-resonant lattice diffraction at *P* = 41 GPa.

Our high-pressure, resonant X-ray diffraction methods are not limited to measuring magnetic order. There is, for example, great potential to extend these techniques to exploring orbital ordering under pressure. Although orbital ordering is manifested mainly through resonant diffraction (Wilkins *et al.*, 2003 ▸), it has to be carefully separated from ATS scattering from aspherical charge distributions, which is typically prominent at *K* edges (McMorrow *et al.*, 2001 ▸; Dmitrienko *et al.*, 2005 ▸). Orbital ordering ideally would be explored by probing a direct resonance through the electric dipole channel as it is the dominant virtual transition (McMorrow *et al.*, 2001 ▸; Wilkins *et al.*, 2003 ▸; Dmitrienko *et al.*, 2005 ▸). 5*d* compounds could serve as potential systems of interest for orbital ordering at high pressure, where the behavior can be directly explored at *L* edges using electric dipole (*p*–*d*) virtual transitions. The resonant technique could reveal the existence of diffusive short-range orbital order produced by the application of pressure, similar to the observation at ambient pressure and finite temperature in Pr_0.5_Ca_0.5_MnO_3_ (Zimmermann *et al.*, 1999 ▸), perhaps even leading to systems with orbital liquid behavior. From a technical perspective, resonant charge scattering was demonstrated previously under pressure at the vanadium *K* edge (*E* = 5.468 K eV) to 1.2 GPa (Ohwada *et al.*, 2007 ▸) and recently at the Os *L*_2_ edge (*E* = 12.387 K eV) in Cd_2_Os_2_O_7_ to 41 GPa at *T* = 4 K amid the measurement of the AIAO antiferromagnetic spin order (Wang *et al.*, 2018 ▸). As demonstrated in more detail in Fig. 6 ▸, the charge resonance profile of the (4, 2, 0) order at the Os *L*_2_ edge remains unchanged across a 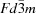 to 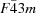 continuous symmetry evolution, before being overwhelmed by the no-longer forbidden lattice diffraction of the 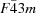 space group.

Our explorations to date have been confined to (*P*, *T*) phase space, with most of the X-ray magnetic diffraction experiments carried out at temperatures at or above 3.5 K. Although it is highly desirable to perform magnetic diffraction at lower temperatures, the current limit is set by the balance of cooling power between the closed-cycle cryostat (∼0.5 W at 4.2 K) and absorbed X-ray energy by the high-pressure sample as a fraction of the total flux (∼20 mW for ∼10^13^ photons s^−1^ at 12 keV), in addition to other thermal loads on the pressure cell. Recently, X-ray resonant magnetic diffraction was performed at the Tm *L* edge (*E* = 8.664 keV) down to 360 mK in a Helium-3 cryostat, with detailed documentation of X-ray heating effects leading to a reduced X-ray flux of ∼10^9^ photons s^−1^ (Francoual *et al.*, 2015 ▸). A further limitation is that many designs of kelvin and sub-kelvin cryostats are limited in the angular range of tilt over which they can operate. The need to probe as wide a range as possible in reciprocal space to gain a full understanding of the lattice, orbital and magnetic evolution under pressure constrains the choice of cooling method to designs such as Gifford–McMahon coolers.

To further expand the parameter space, a natural addition would be a vector magnetic field **H**, leading to a much broader (**H**, *P*, *T*) phase space. It has been shown that a diamond-anvil cell can be made fully out of non-ferromagnetic materials with very low magnetic permeability (Palmer *et al.*, 2015 ▸). The major remaining technical challenge is to manufacture a superconducting magnet, presumably using high-*T*_c_ cuprate ribbons, that would be thermally anchored at the first cooling stage of the Gifford–McMahon cryostat (∼50 K). In this way, the magnet would be thermally decoupled from the sample and pressure cell, yet still able to provide a magnetic field that could vary in both magnitude and direction relative to the single-crystal sample. Such a design could potentially lead to studies of collective spin behavior such as skyrmions under pressure.

The advent of fourth-generation synchrotron radiation sources (Hettel, 2014 ▸) is expected to significantly improve the counting statistics of high-pressure X-ray magnetic diffraction, making measurements more time-efficient and potentially routine, albeit more difficult to cool to cryogenic temperatures. Furthermore, there is a new synchrotron-radiation-based research field of resonant magnetic diffuse scattering, which, so far, has only been demonstrated at ambient temperature (Chun *et al.*, 2015 ▸). While orbital diffuse scattering under pressure is a possibility at a third-generation synchrotron radiation source, probing magnetic correlations and fluctuations under pressure using resonant magnetic diffuse scattering would probably require state-of-the-art instrumentation at fourth-generation synchrotron sources. Nevertheless, it would be an exciting research frontier to explore both quantum criticality and spin dynamics under high-resolution tuning conditions, providing a natural extension from X-ray magnetic diffraction at ambient pressure using second-generation synchrotron sources, and under GPa high pressure using third-generation synchrotron sources.

## Figures and Tables

**Table 1:** Sample-chamber parameters for various pressure ranges These parameters are designed for the condition of using a pair of wide-perforated Boehler anvils, together with a methanol:ethanol 4:1 mixture as the pressure medium. Pressure is expected to be increased only at low temperature (typically no higher than 25 K) using a helium diaphragm, in order to reduce the thermal expansion of the pressure medium and any thermal weakening of the gasket strength. The pressure is calibrated by the equation of state for silver (Ag) at *T* = 4 K using two parameters (bulk modulus *B* = 108.85 GPa, and *B*′ = d*B*/d*P* = 5.7).

Diamond culet size (µm)	Gasket materials	Initial shim thickness (µm)	Pre-indented thickness (µm)	Hole diameter (µm)	Targeted pressure (GPa)
800	Stainless Steel 301	305	162	390	18
700	Rhenium	255	156	330	25
600	Rhenium	255	141	280	35
550	Rhenium	255	121	240	42
